# Omission responses in local field potentials in rat auditory cortex

**DOI:** 10.1186/s12915-023-01592-4

**Published:** 2023-05-30

**Authors:** Ryszard Auksztulewicz, Vani Gurusamy Rajendran, Fei Peng, Jan Wilbert Hendrik Schnupp, Nicol Spencer Harper

**Affiliations:** 1 Center for Cognitive Neuroscience Berlin, Free University Berlin, Berlin, Germany; 2 Dept of Neuroscience, City University of Hong Kong, Hong Kong, Hong Kong S.A.R.; 3 Dept of Physiology, Anatomy and Genetics, University of Oxford, Oxford, UK

**Keywords:** Predictive processing, Auditory processing, Omission responses, Electrophysiology, Auditory cortex

## Abstract

**Background:**

Non-invasive recordings of gross neural activity in humans often show responses to omitted stimuli in steady trains of identical stimuli. This has been taken as evidence for the neural coding of prediction or prediction error. However, evidence for such omission responses from invasive recordings of cellular-scale responses in animal models is scarce. Here, we sought to characterise omission responses using extracellular recordings in the auditory cortex of anaesthetised rats. We profiled omission responses across local field potentials (LFP), analogue multiunit activity (AMUA), and single/multi-unit spiking activity, using stimuli that were fixed-rate trains of acoustic noise bursts where 5% of bursts were randomly omitted.

**Results:**

Significant omission responses were observed in LFP and AMUA signals, but not in spiking activity. These omission responses had a lower amplitude and longer latency than burst-evoked sensory responses, and omission response amplitude increased as a function of the number of preceding bursts.

**Conclusions:**

Together, our findings show that omission responses are most robustly observed in LFP and AMUA signals (relative to spiking activity). This has implications for models of cortical processing that require many neurons to encode prediction errors in their spike output.

## Background

At least since the times of von Helmholtz [1], prediction has been proposed as important to perception, and many principled models of cortical function have prediction of current or future sensory input as a central component [2–6]. Efficient prediction of future sensory inputs may facilitate action guidance and sensory feature extraction [7] and hence may be a key principle governing sensory neural systems, arguably explaining many of their features [6]. Thus finding neural representations of predictions - or of prediction errors, resulting from deviations of sensory inputs from their predictions - has been a recent area of intense research focus. Central to these investigations have been oddball paradigms, in which a sequence of expected stimuli is replaced by an unexpected stimulus [8, 9]; these paradigms have provided insights into neural prediction using behavioural [10], EEG/MEG [8, 11, 12], and in vivo neurophysiological measurements [13–15]. However, rather than altering the expected stimulus, it can instead be omitted, enabling observations of the form and timing of predictive signals that are not confounded by processing of incoming stimuli [16]. Although omission responses have often been reported using measurements of neural activity in humans that are non-invasive [17–24] or from the cortical surface [25, 26], there has been little investigation at a detailed level by using penetrating electrodes.

**Fig. 1:**
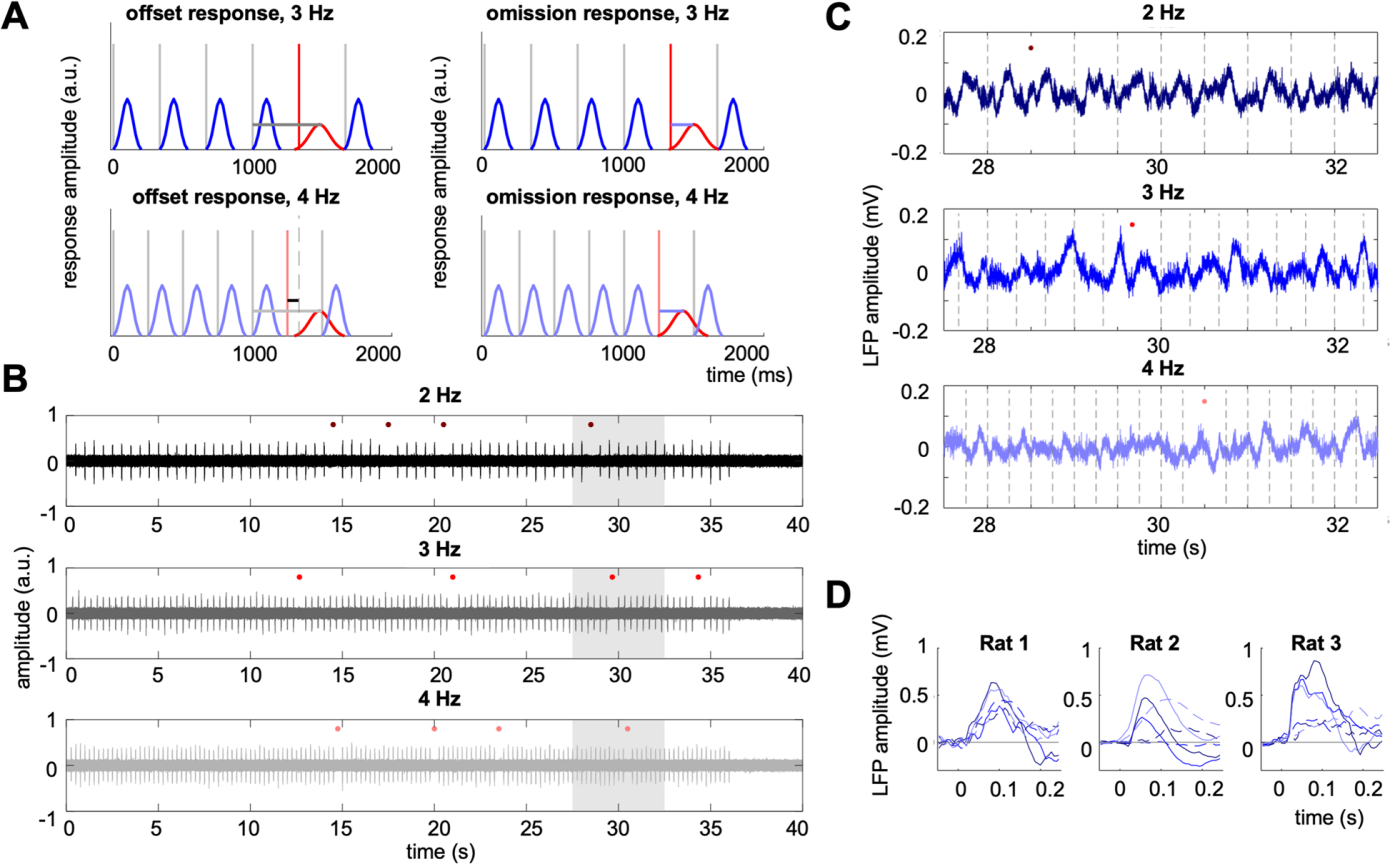
**A** Schematic representing two possible types of neural responses that could occur to omissions of rhythmically presented stimuli. Vertical lines represent stimuli presented at 3 Hz (dark grey lines, upper plots), 4 Hz (light grey lines, lower plots), or omitted (red lines). Hypothetical responses to noise bursts are plotted in blue. Left: offset response, whose latency is locked (as marked by the grey horizontal line) to the preceding stimulus rather than to the omitted stimulus. Right: true omission response, whose latency should be locked (as marked by the grey horizontal line) to the omitted stimulus rather than to the preceding stimulus. When analysing response latencies relative to the omitted stimulus, true omission responses should show the same latency relative to the expected time of the omitted response for both 3 and 4 Hz, while offset responses should show a latency shift between 3 and 4 Hz (lower left plot, difference between solid red vertical line, showing expected 4 Hz offset, and dashed grey vertical line, showing expected 3 Hz offset; the difference is marked by the upper horizontal line). **B **Example stimulus waveforms for 2 Hz (upper plot), 3 Hz (middle plot), and 4 Hz (lower plot) sequences. Red dots denote omitted stimuli. Shaded area denotes the time segment for which raw local field potentials are plotted in C. **C** Example local field potentials from a representative electrode. Dashed vertical lines denote presented stimuli. Red dots denote omitted stimuli. **D** Examples of LFP responses averaged over either bursts or omissions, plotted for a representative channel for each rat. Solid lines: average stimulus-evoked responses; dashed lines: average omission responses; colours as above

Non-invasive human studies with steady trains of stimuli suggest that responses to omitted stimuli peak later than stimulus-evoked responses and are often reported to have lower amplitudes [27], but see [28]. Crucially, these omission responses have a broad frequency spectrum [20] and are time-locked to the expected onset of an omitted stimulus [23]. This differentiates them from offset responses, which are time-locked to the end of each stimulus (or fast stimulus trains) rather than to the onset of the expected but omitted stimulus (Fig. 1A) [29]. Offset responses can have substantial latencies and durations [30], and thus could be mistaken for omission responses if their latency coincided with the period of the stimulus train. For this reason, it is important to use a range of stimulus presentation rates when looking for omission responses.

Early non-invasive human studies suggested that omission responses can be observed only for relatively fast stimulus presentation rates, well above 5 Hz [17], which was interpreted as a limited window of temporal integration. However, mounting evidence demonstrates that omission responses can also be observed in awake humans following longer inter-stimulus intervals, in the sub-second [26, 31] and supra-second range [32]. These omission responses at different time scales may be differentially influenced by cognitive factors: for instance, omissions following short ISIs (periodicity > 5 Hz) have been suggested to be elicited entirely automatically, while slower time scales (periodicity < 2 Hz) may be modulated by attention [23, 29, 33], but see [25].

The attentional involvement in omission-related activity has also been found in extracellular signals recorded in the auditory cortex of trained awake macaques attending to auditory streams presented at rates of ~ 2 Hz [34]. However, these findings in non-human primates, together with findings in humans described above suggesting that omission responses to stimuli omitted at slow time scales require attention, are seemingly inconsistent with invasive cellular-scale studies in rodent models, where omission-related activity has also been found in anaesthetised animals exposed to very slow (≤ 0.5 Hz) isochronous sequences. In the latter case, omission-related activity has been reported in the non-lemniscal thalamus of guinea pigs using intracellular recordings [35] and in the auditory cortex of mice [36, 37], although necessitating a large number of preceding standards. However, the last two studies used calcium imaging, which is characterised by slowly decaying calcium signals, rather than direct electrophysiological recordings of neural activity. Hence, it is largely unknown if (1) omission responses are homologous across species, such that they can be found in passively listening rodents at the faster time scales typical for human omission responses, and (2) how these fast omission responses are instantiated at a cellular scale.

Here, we used penetrating microelectrodes to record local neural population activity from the auditory cortex of anaesthetised rats to trains of noise bursts at 2, 3 or 4 Hz burst rates, with noise bursts occasionally omitted at random. By combining fast presentation rates (associated with higher temporal precision [38]) with the excellent temporal resolution of electrophysiological recordings, we could analyse the relative latency of omission responses with high precision. We observed that the local field potentials and analogue-multiunit activity at the majority of recording sites showed a response just after the time point when a stimulus was expected but omitted. These omission responses had a fixed latency relative to the expected onset regardless of stimulus rate, indicating true omission responses rather than offset responses. The omission signals increased as a function of the number of preceding noise bursts, suggesting that they might be modulated by the strength of previously formed predictions. However, such omission responses were not apparent in the single-unit or multi-unit responses. Together this suggests that prediction or prediction error might be represented in signals reflecting inputs to the auditory cortex or dendritic activity, but its representation may be rare or non-existent in the spiking activity of auditory cortical neurons, at least under the anaesthetised and high-burst-rate condition we examined. This may limit the space of possible models of the cortex involving prediction.

## Results

### Putative omission responses were observed in LFP and AMUA signals, but not in single/multi-unit spiking activity

**Fig. 2:**
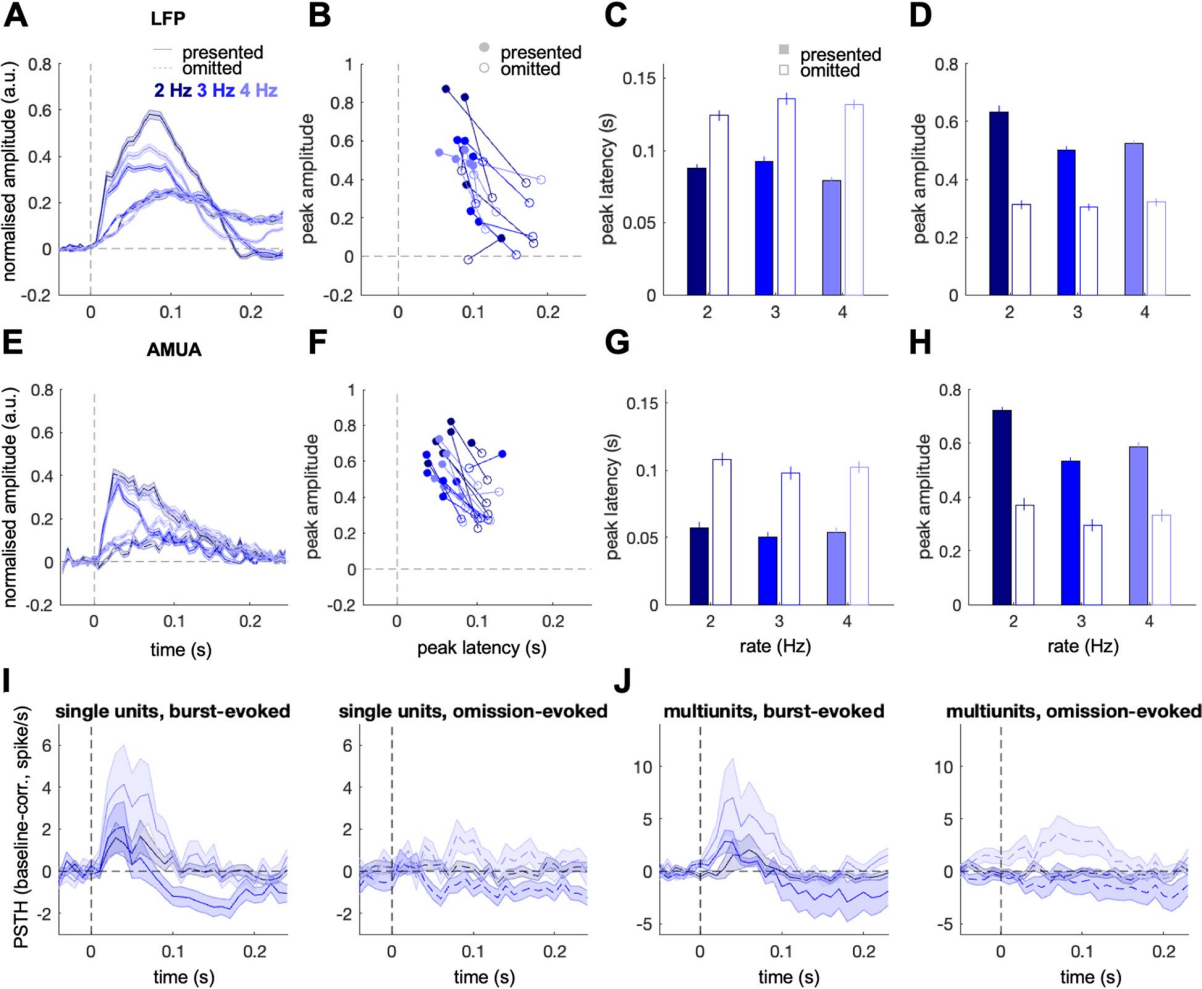
**A**–**D** Local field potential (LFP) responses to presented vs. omitted stimuli, normalised to pre-stimulus baseline (*n* = 6 penetrations). **E**–**H** Analogue multiunit activity (AMUA) responses to presented vs. omitted stimuli, normalised to pre-stimulus baseline (*n* = 6 penetrations). **A**, **E **Time courses of responses (normalised to pre-stimulus baseline) evoked by presented stimuli (solid lines) relative to stimulus presentation (noise bursts immediately preceding omissions), and by stimulus omissions (dashed lines) relative to the expected but omitted stimulus onset. Dark blue, blue, and light blue lines correspond to stimulus presentation rates of 2, 3, and 4 Hz. Shaded areas represent standard error of the mean (SEM) across channels. **B**, **F** Peak amplitudes (*Y*-axis) and latencies (*X*-axis) of each penetration, averaged across analysed channels. Filled circles: stimulus-evoked responses; empty circles: omission responses; colours as above. **C**, **G** Peak amplitude comparison of stimulus-evoked (filled bars) and omission responses (empty bars) across the three stimulus presentation rates. Error bars represent SEM across channels. Please note that single channels are presented in B and F. **D**, **H** Peak amplitude comparison of stimulus-evoked vs. omission responses across the three stimulus presentation rates (filled/empty bars as above). Error bars represent SEM across channels. **I** Baseline-corrected peristimulus time histograms (PSTHs), quantifying single-unit spiking activity responses to noise bursts (left panel) and omitted stimuli (right panel). Colours as in A and E. Shaded areas represent SEM across units. **J** Multiunit PSTHs. Legend as in I

We recorded a total of 6 penetrations from 3 rats using multi-electrode eight-shank probes with 8 electrodes (channels) along each shank. Anaesthetised naive rats were exposed to trains of noise bursts presented at an isochronous rate of 2, 3 or 4 Hz, with a random subset of 5% of noise bursts omitted from each train (Fig. 1; see the ‘ Methods’ section). The probes penetrated perpendicularly through the auditory cortex to record across its full depth. This resulted in a total of 384 channels recorded, with 64 channels per penetration. Data were analysed in two broad frequency bands, including lower (0.1–75 Hz; LFP analysis) and higher (300–6000 Hz; AMUA analysis) frequencies, which have been proposed to be predominantly sensitive to summed inputs and local outputs of neural populations respectively [39, 40], but see [41]. The data were additionally spike-sorted to yield 43 single units and 70 multi-units that passed a response reliability criterion (see the ‘Methods’ section). Population local field potentials (LFP), analogue multiunit activity (AMUA), and spiking activity following presented and omitted bursts are shown in Fig. 2.

First, to quantify the proportion of channels showing significant activity in the burst-evoked time window (0–250 ms relative to burst onset) or in the omission time window (0–250 ms relative to expected but omitted burst onset), for each channel we pooled single-trial time-average amplitude estimates across the three burst rates and entered them into one-sample *t*-tests. Single trials were defined as responses to single bursts (immediately preceding omitted stimuli), or expected but omitted bursts. Single-trial responses were baseline-corrected (see the ‘ Methods’ section). To avoid signals from electrodes with different polarity cancelling each other out, LFP traces from those channels which showed a negative burst-evoked peak amplitude (averaged across presentation rates) were sign-flipped. In the LFP analysis (Fig. 2A), on average, 35.16 channels (SEM 9.60, corresponding to 54.95% ± 15% channels) per penetration showed significant responses to both presented sounds and sound omissions (averaged across rates), while in the AMUA analysis (Fig. 2E), on average, 21.33 channels (SEM 4.82, corresponding to 33.33% ± 7.53% channels) per penetration showed significant responses to both presented sounds and sound omissions (one-sample *t*-tests; in both LFP and AMUA, p_FDR_ < 0.05, false discovery rate corrected; [42]). Additionally, a number of channels responded only to presented sounds (LFP: 24.74% ± 15.26%; AMUA: 38.54% ± 9.06%), and a smaller proportion of channels responded only to omitted sounds (LFP: 9.64% ± 7.86%; AMUA: 4.16% ± 1.83%). No responses to presented or omitted sounds were recorded in the remaining channels (LFP: 7.81% ± 3.56%; AMUA: 25.26% ± 8.91%).

In contrast to LFP and AMUA signals, the analysis of spiking activity in 113 single and multi unit responses did not yield consistent omission responses. Among our 43 single units (with 11 units assigned to superficial channels, 10 to intermediate channels, and 22 to deep channels; see the ‘ Methods’ section), none showed a significant omission response relative to pre-stimulus baseline while correcting for multiple comparisons (all p_FDR_ > 0.05; Fig. 2I), with only 3/43 units showing post-omission PSTH higher than the null distribution (generated based on 1000 simulated PSTHs; see the ‘ Methods’ section) at an uncorrected *p* < 0.05. Similarly, among 70 analysed multiunits (with 18 units assigned to superficial channels, 15 to intermediate channels, and 37 to deep channels), none showed a significant omission response (all p_FDR_ > 0.05; Fig. 2J), and 9 units had the post-omission PSTH survive the uncorrected threshold of *p* < 0.05. While a visual inspection of both single- and multiunit activity did indicate relatively robust activity in the post-omission period for the 4 Hz burst rate, this activity started before the expected (but omitted) burst, suggesting that it corresponds to an offset response rather than to a true omission response. No such activity was observed for the slower burst rates (2 and 3 Hz).

### Amplitudes and latencies of omission-evoked responses

Since we only observed omission responses in the LFP and AMUA data, we further analysed these two signal types. Our first aim was to compare the latencies and amplitudes of neural responses to presented and omitted sounds (Fig. 2B, F), and the analyses that follow are thus focused on channels displaying significant burst-evoked and omission-evoked responses. In both LFP and AMUA analyses, single-channel data (peak amplitude or peak latency values) were entered into a mixed-effects model with two fixed-effects factors (stimulus type: burst vs. omission; presentation rate: 2, 3, and 4 Hz) and one random-effects factor (penetration). In both LFP and AMUA signals, there was a significant difference between responses evoked by burst omissions and preceding burst presentations in terms of the peak amplitude (main effect of stimulus type; LFP: F_1,1176_ = 13.55, *p* = 0.021, Fig. 2C; AMUA: F_1,732_ = 41.86, *p* < 0.001, Fig. 2G), with omission-evoked responses having lower peak amplitudes than burst-evoked responses. Additionally, in the AMUA but not the LFP, there was a trend towards a main effect of presentation rate (F_2,732_ = 3.8, *p* = 0.0563). Qualitatively, the responses to bursts presented at 3 Hz were weaker than to those presented at 2 Hz and 4 Hz, which may be explained by a form of long-term adaptation: if responses adapt to burst trains of a particular frequency as well as adjacent frequencies, 3 Hz burst responses would be expected to adapt to all rates (2–4 Hz), as opposed to 2 Hz and 4 Hz burst responses which may only adapt to their own rate and the adjacent 3 Hz. Nevertheless, the interaction between presentation rate (2, 3, and 4 Hz) and stimulus (omission vs. burst) was not significant in either LFP or AMUA analysis (both *p* > 0.18), suggesting that the relative strength of omission responses does not depend on presentation rate.

There was also a significant difference in peak latency between responses evoked by omitted and presented stimuli in LFP and AMUA (LFP: F_1,1176_ = 8.84, *p* = 0.04, Fig. 2D; AMUA: F_1,732_ = 38.21, *p* < 0.001, Fig. 2H), with omission responses peaking later than burst-evoked responses. Crucially, neither the main effect of presentation rate nor the interaction between presentation rate and stimulus were significant (both AMUA and LFP analysis: *p* > 0.3), suggesting that the latency of omission responses is locked to the onset of an expected but omitted noise burst, and is not modulated by presentation rate. This is consistent with omission responses rather than offset responses.

**Fig. 3:**
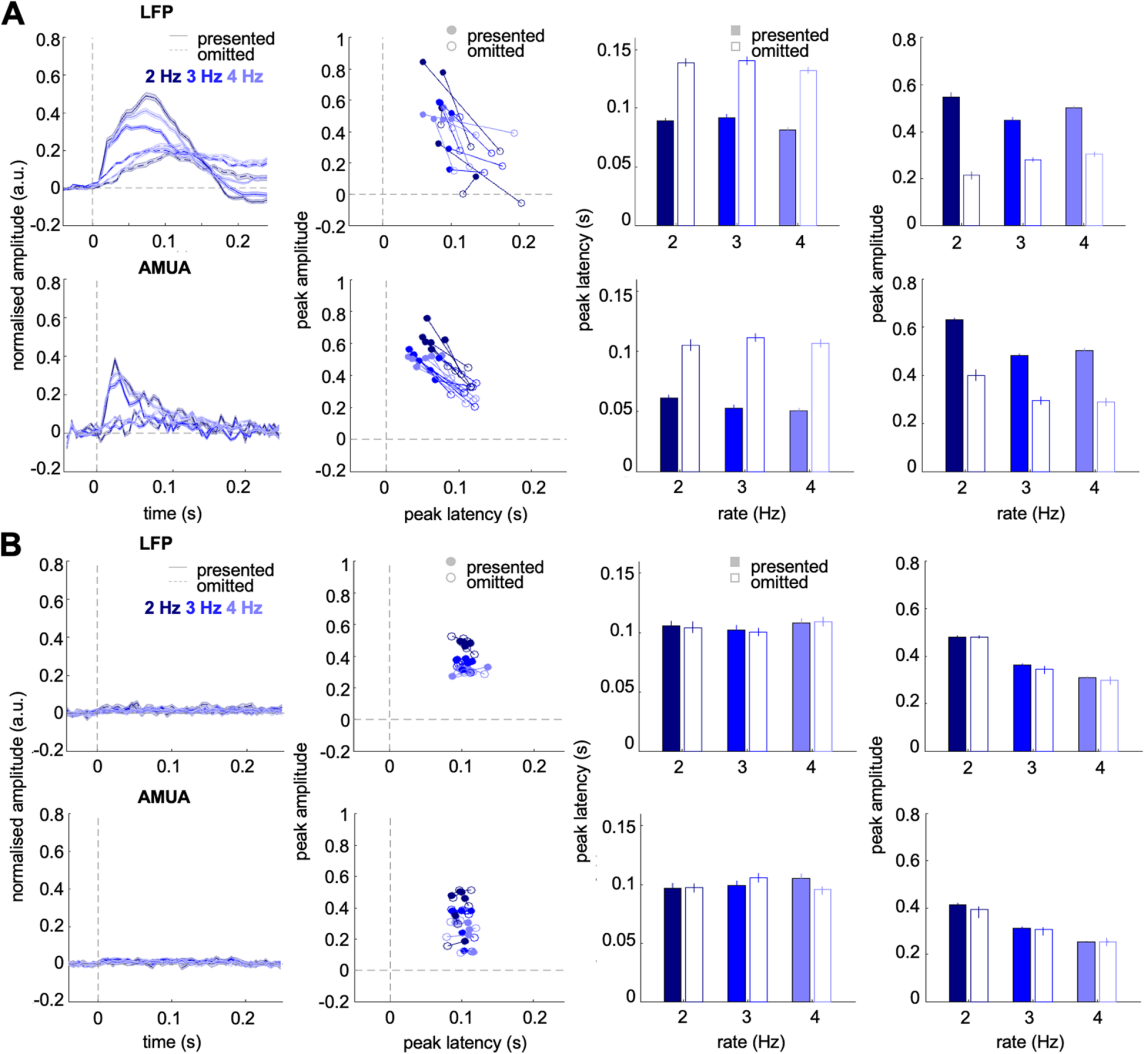
**A** LFP (upper panels) and AMUA (lower panels) activity analysed for all channels, with no channel selection criteria (*n* = 6 penetrations). Figure legend as in Fig. 2A–H. **B** LFP (upper panels) and AMUA (lower panels) activity analysed for time-shuffled data. Figure legend as in Fig. 2A–H

The same pattern of results was replicated in a control analysis, in which all channels were included (rather than only those showing a significant response to bursts and omissions; Fig. 3A). In the LFP data, both amplitude and latency differed between burst-evoked and omission-evoked responses (amplitude: F_1,1896_ = 8.99, *p* = 0.03; latency: F_1,1896_ = 10.9, *p* = 0.021) but the main effects of presentation rate and the interaction effects between presentation rate and stimulus were not significant (*p* > 0.15). In the AMUA data, beyond the main effect of stimulus on both amplitude (F(1,2232) = 36.612, *p* = 0.002) and latency (F_1,2232_ = 60.35, *p* < 0.001), we also found a significant main effect of presentation rate on amplitude (F_2,2232_ = 26.29, *p* < 0.001) but not on latency (*p* > 0.3). The interaction effects between presentation rate and stimulus were not significant (*p* > 0.3). Thus importantly, the latency for the LFP or AMUA omission-evoked responses had no significant dependence on presentation rate, again suggesting locking to omission onset.

**Fig. 4:**
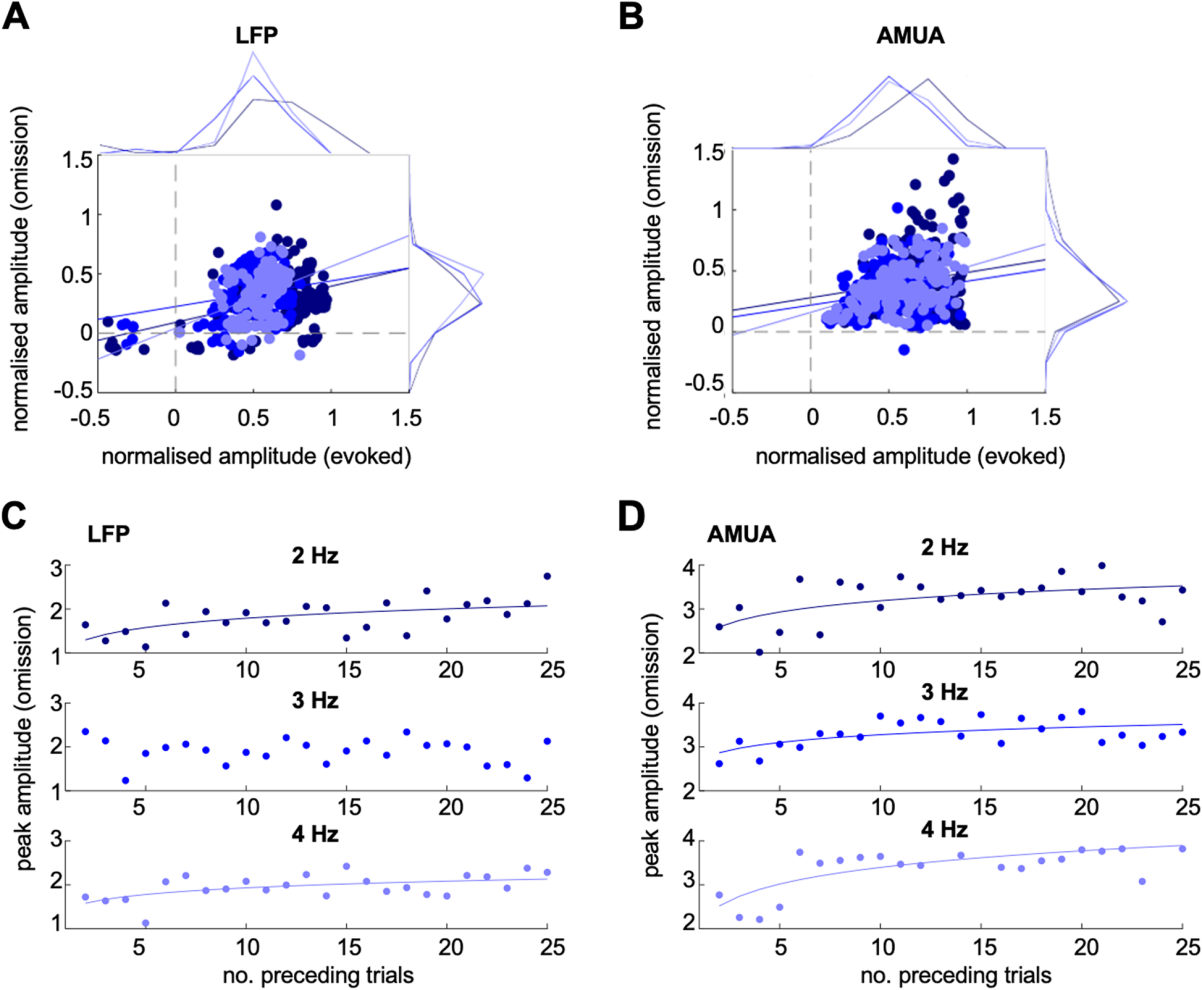
**A** Scatterplot and marginal histograms of peak LFP amplitude relationship between stimulus-evoked (*X*-axis) and omission responses (*Y*-axis) for each presentation rate (dark blue: 2 Hz, blue: 3 Hz, light blue: 4 Hz). Solid lines denote regression slopes. **B **Scatterplot and marginal histograms of peak AMUA amplitudes; legend as above.** C** Peak LFP amplitudes of omission responses as a function of the number of preceding noise bursts (dark blue: 2 Hz, blue: 3 Hz, light blue: 4 Hz). Solid lines denote significant regression slopes. **D **Peak AMUA amplitudes of omission responses as a function of the number of preceding noise bursts. Legend as above

A further control analysis was performed (Fig. 3B) to confirm that the omission responses identified above were not due to random noise fluctuations in the post-omission time window. To this end, we shuffled the signal amplitudes in each single-trial response (i.e. responses to each individual expected but omitted burst) over time points in the entire time window (-100–250 ms relative to expected burst onset) and analysed the data in the same manner as in the main analysis. We reasoned that, if omission responses are due to noise fluctuations, shuffling data over time points should not affect the peak amplitudes. Conversely, if omission responses reflect neural activity following an expected but omitted stimulus, shuffling data will abolish any omission-locked activity peaks. The analysis of time-shuffled LFP data revealed no main effect of stimulus on signal amplitude (*p* = 0.0838) and no interaction between stimulus and presentation rate (*p* = 0.6405), but a main effect of presentation rate (F_2,2232_ = 580.57, *p* < 0.001). The analysis of LFP response latency revealed no main or interaction effects (*p* > 0.5). Similarly, the analysis of time-shuffled AMUA data revealed no main effect of stimulus on signal amplitude (*p* = 0.2104) and no interaction between stimulus and presentation rate (*p* = 0.2974), but a main effect of presentation rate (F_2,2232_ = 35.25, *p* < 0.001). The analysis of AMUA response latency revealed no main or interaction effects (*p* > 0.25). Please note that each point in the scatter plots shown in Fig. 4B is based on the average of single-channel maxima (which can occur anywhere in the analysed time window, and is therefore non-zero), while the average time course does not show any robust peaks (since their latency is not consistent over the analysed time window, based on shuffled data).

To test whether omissions can be attributed to the same channels which show evoked responses, we analysed the correlations between the peak amplitudes of evoked and omitted responses across channels. This analysis revealed significant correlations for both types of analyses (LFP, AMUA) and all presentation rates (2, 3, and 4 Hz). In the LFP analysis (Fig. 4A), the correlation coefficients were r = 0.4465, *p* < 0.001 (2 Hz); *r* = 0.2185, *p* = 0.002 (3 Hz); *r* = 0.5401, *p* < 0.001 (4 Hz). In the AMUA analysis (Fig. 4B), the correlation coefficients were *r* = 0.1515, *p* = 0.0433 (2 Hz); *r* = 0.1947, *p* = 0.0091 (3 Hz); and *r* = 0.4367, *p* < 0.001 (4 Hz).

### Buildup of omission responses over time

Next, we explored whether the size of omissions responses could depend on the number of bursts preceding it. For each burst rate, we plotted the amplitude of omission responses (averaged over channels) as a function of the number of preceding noise bursts since the previous omission. We then made a linear fit to the dependence of the amplitude on the log of the number of preceding bursts; we applied the log due to the exponential nature of adaptation effects [43]. Measuring the significance of these fits revealed a significant increase of LFP omission responses as a function of the number of preceding bursts (Fig. 4C) for all but one burst rate (2 Hz: *r* = 0.5516, *p* = 0.0052; 3 Hz: *r* =  − 0.1448, *p* = 0.4996, n.s.; 4 Hz: *r* = 0.5292, *p* = 0.0078). All significant correlations survived Bonferroni correction for multiple comparisons. In the AMUA analysis (Fig. 4D), the correlation coefficients were significant for all analysed rates (2 Hz: *r* = 0.5306, *p* = 0.0076; 3 Hz: *r* = 0.5522, *p* = 0.0051; 4 Hz: *r* = 0.6998, *p* < 0.001). All significant correlations survived Bonferroni correction for multiple comparisons. It should be noted that, in this analysis, omission peaks were extracted at a single-trial level (and then averaged across trials preceded by a specific number of bursts), while in Figs. 2 and 3, omission peaks were extracted at a trial-average level, resulting in a relatively lower range of amplitudes. Taken together, these findings are consistent with it taking time for the brain to build a temporally-local model of the standard stimulus (noise burst), generate predictions, and signal errors (omission responses).

### Cortical depth of omission-evoked responses

To analyse the approximate cortical depth of omission-evoked responses, we transformed the LFP data into current source density (CSD) profiles to minimise the effect of volume conduction and obtain more precise estimates of local synaptic current flow [34]. We tested whether the relative strength of omission and evoked responses shows any differences in depth and whether these differences depend on the type of analysed responses (CSD vs. concomitant AMUA), by grouping responses into three levels by channel depth: superficial, intermediate, and deep (see Methods). To quantify the relative strength of omission responses, per channel, we calculated the omission response index (see the ‘Methods’ section) by scaling the omission response amplitude to the average overall response amplitude (omission and evoked responses combined).

**Fig. 5:**
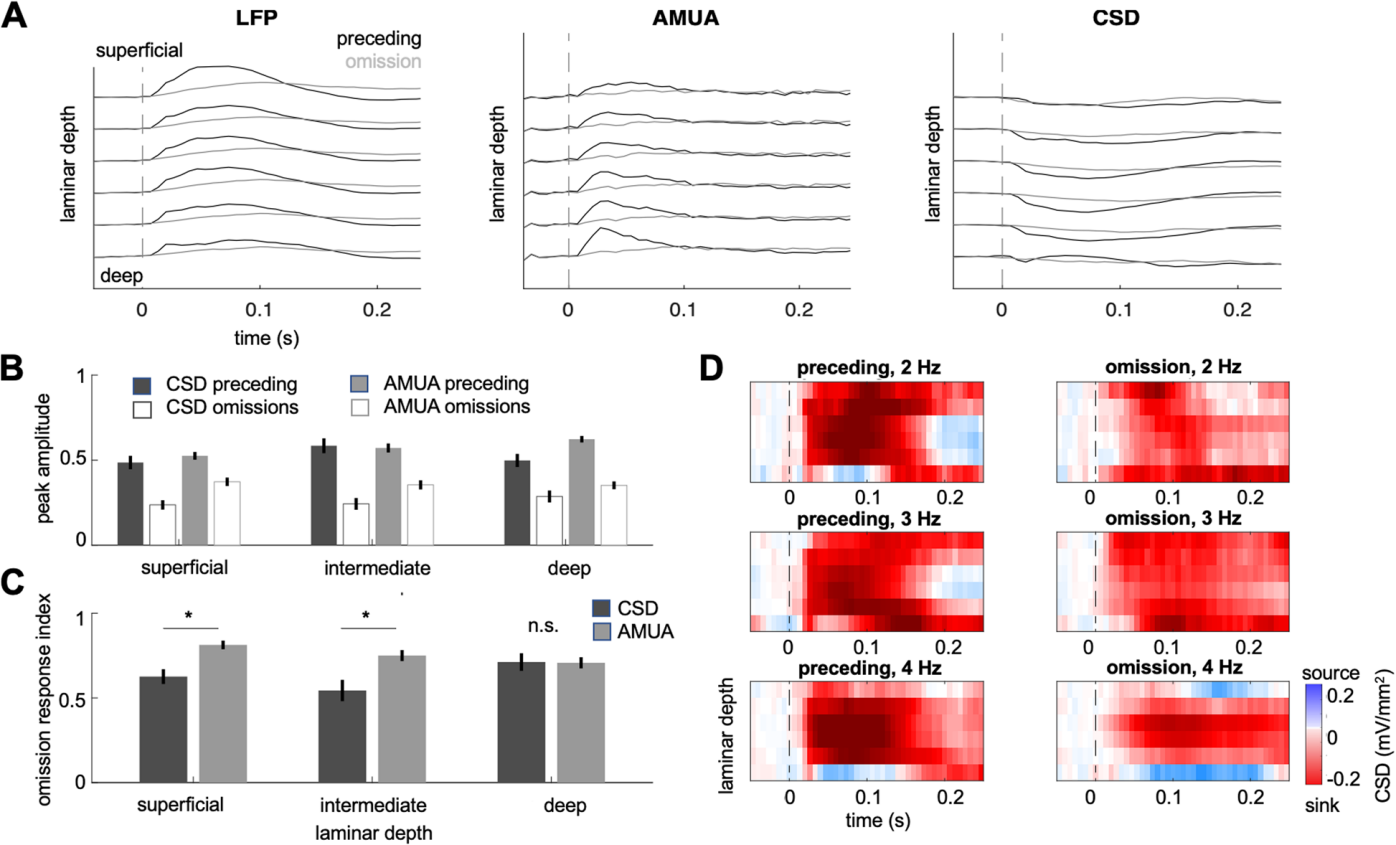
**A** LFP, AMUA, and CSD time courses across channel depth (*n* = 6 penetrations). The six pairs of lines show preceding burst and omission responses at the 2nd to the 7th electrode going down, the spacing of the electrodes was 200 μm. **B** Cortical depth profile of omission responses: peak CSD and AMUA amplitudes plotted for stimulus-evoked (solid bars) and omission responses (empty bars) for CSD (dark grey) and AMUA (light grey). Error bars represent SEM across channels. **C** Cortical depth of the omission response index (see the ‘ Methods’ section) for CSD (dark grey) and AMUA (light grey). Asterisk marks a significant difference between CSD and AMUA (*p* < 0.05, Bonferroni corrected across cortical depth). Error bars represent SEM across channels. **D **CSD maps per burst rate, plotted separately for stimulus-evoked and omission responses. Cortical depth as in A

Overall, the omission response index was significantly higher for AMUA than for CSD (F_1,200_ = 8.53, *p* = 0.0046). Interestingly, we also found a significant interaction between signal type (AMUA vs. CSD) and channel depth (F_2,200_ = 4.23, *p* = 0.0159). Given this interaction, we then compared the resulting omission response indices between response types (CSD vs. AMUA), separately for each channel depth (Fig. 5). This analysis revealed that AMUA omission responses were relatively stronger than CSD omission responses in the superficial channels (two-sample t-test: t_71_ = 3.89, *p* < 0.001) and in the intermediate channels (t_66_ = 3.21, *p* = 0.002), but not in the deep channels (t_82_ = 0.08, *p* = 0.938).

When analysing AMUA and CSD as a function of depth individually, we found no significant difference between channel groups in the case of AMUA omission response indices (F_2,74_ = 1.82, *p* = 0.1693), but a significant difference between channel groups in the case of CSD (F_2,135_ = 3.69, *p* = 0.0274). A post hoc analysis of the latter finding showed a significant decrease of CSD omission response indices in the superficial vs. deep channels (t_94_ = 2.41, *p* = 0.0176), but not in the other pairwise comparisons (*p* > 0.13).

## Discussion

We have identified robust omission responses in the auditory cortex of anaesthetised naive rats, suggesting that omission detection is an automatic brain function. These omission responses are temporally locked to the anticipated stimulus onset (but not to the onset of the preceding standard stimulus), consistent with their interpretation as representing a predictive signal rather than an offset response. However, they are observable only in the local field potentials and analogue multiunit activity, but not in spiking activity, suggesting that they might be mediated by mechanisms other than classical spiking activity of projection neurons. In the following, we discuss the response characteristics and functional significance of omission responses.

### Omissions responses at the cellular level in the auditory cortex

The cellular-level omission responses that we observe in the auditory cortex bear similarities with more gross non-invasive recordings of omission responses from the auditory cortex of humans. The omission response we observed had lower amplitudes and longer latencies than stimulus-evoked responses, consistent with previous non-invasive results in humans [20, 23, 27]. Non–invasive measurements of neural activity in humans have identified omission responses for a wide range of stimulus presentation rates, with ISIs as short as 200 ms [17] or as long as 2 s [32], which spans the range we examined.

There have been a few studies examining omission-related responses at a cellular level in the auditory cortex. Recent studies using calcium imaging in the auditory cortex of awake and anaesthetised naive mice have reported echo responses (following sequence termination) for long ISIs in the range of 2–4 s [36, 37], but they did not see them at a faster rate of 1 s ISI—unlike in our results, which show omissions responses for even faster rates of 0.25–0.5 s ISI. The protocol for these calcium imaging studies was very different from ours, having no omissions reoccurring during the stimulus train, and the only omission being the end of a single stimulus train. Furthermore, neural activity measured using calcium imaging is characterised by much longer time constants relative to electrophysiological measurements, which makes it a suboptimal method of imaging time-courses of omission responses at faster presentation rates. Thus, our study is the first to show temporally specific omission responses in the auditory cortex of naive animals at presentation rates typical for most previous non-invasive human studies of neural activity [20, 22–24], and for occasional omissions in sequences of standard stimuli. The omission signals observed in our study increased as a function of the (log) number of preceding noise bursts, suggesting that they might be modulated by the strength of predictions, based on the number of preceding standard stimuli [43]. Omission responses derived from both LFPs and AMUA at all three rates show this pattern of results, with the exception of LFP omission responses to missing stimuli presented at 3 Hz. The latter (null) finding, as well as the overall weaker amplitude of responses to stimuli presented at 3 Hz (see Fig. 2D, H), may be explained by a form of additional long-term adaptation in broadly tuned populations due to previous stimulation in neighbouring burst rates (2 Hz; 4 Hz). For the 3 Hz rate, such general adaptation effects may be arguably stronger than for the “edge” rates of 2 Hz and 4H z, possibly resulting in attenuated response amplitudes, and weaker effects attributable to previous stimuli within the 3 Hz sequences. Another possibility is that our effects may be due to a non-monotonic effect of presentation rate on evoked response amplitudes, as it has been shown in humans that auditory evoked N1 amplitudes first decrease as a function of increasing presentation rate (up to ~ 3 Hz), and then increase for even faster rates [44]. Nevertheless, the gradual buildup of omission signals over time observed for the remaining rates is consistent with the literature on mismatch responses following repetition suppression to consecutive standards [45, 46], as well as with a recent study showing a gradual buildup of predictive information (decodable from auditory cortex in anaesthetised rats) as a function of the number of preceding predictable stimuli [47].

### Omission responses under passive listening

While observing omission-related activity in naive animals suggests that it is a relatively automatic neural response, this conclusion is further reinforced by the fact that we measured neural activity from anaesthetised animals. This is consistent with previous reports that, in awake humans, omission responses can be observed under passive listening, with no attentional involvement [23]. To the best of our knowledge, no study so far has investigated omission responses in humans under anaesthesia or in disorders of consciousness. However, another type of neural response commonly associated with prediction error signals—namely, a mismatch response to deviant tones—is observed also under anaesthesia [48] and in the vegetative state [49]. In a previous study which compared omission-related activity in awake and anaesthetised mice [36], omission responses were found to be only slightly more prevalent in the awake state (~ 21% neurons in the superficial layers of the auditory cortex showing omission responses in the awake state, compared to ~ 15% neurons under anaesthesia)—however, as noted above, this was at a slower stimulus rate than in our study. Taken together, our and previous results suggest that rudimentary temporal predictions, which result in omission responses to stimulus absence in a rhythmic context, are generated automatically, without the involvement of attention (behavioural relevance) or wakefulness.

Nevertheless, the precise neural computations underlying an omission response remain to be elucidated. For instance, while an omission response may reflect a prediction error due to an unfulfilled prediction of a particular stimulus occurring at a particular time, it may also reflect a sequence-stopping response, marking the end of a temporally predictable sequence (which can only be detected after the onset of the first omitted stimulus) but independent of content-based expectations. Furthermore, at least in isochronous sequences with repeated identical standard tones, omission responses may also be explained by adaptation mechanisms without the involvement of canonical predictive processing [50]. While previous studies in humans showed that the unfulfilled predictions of stimulus contents can be decoded from neural omission responses [51], a recent attempt to induce similar stimulus-specific omission responses in naive anaesthetised rats was not successful [47]. However, both of these studies relied on tone sequences which were more complex than repeated identical standard tones. To disentangle tone-specific omission responses from sequence-stopping responses, it would be necessary to manipulate the content expectations of the sequences. While it has been recently shown that the neural processing of sequences is enhanced in rats when they are previously trained to discriminate the sequences using operant conditioning [52], it remains to be tested whether anaesthetised, passively listening rats are a good model for studying omission responses when they are previously trained on sound sequences, rather than naive.

Previous studies have occasionally interpreted omission responses as resulting from neural entrainment [34, 36, 37]. According to the neural entrainment hypothesis, an influential model of the modulation of neural activity in sensory cortices by rhythmic stimulus presentation [53], isochronous sound delivery should gradually increase the phase locking of low-frequency activity in the auditory cortex, at a frequency specific to the stimulus presentation rate [34], resulting in strong phase-locking not only at the times of actual stimulus presentations, but also around the times when omitted stimuli would be expected. The omission responses we observe are not particularly consistent with the neural entrainment hypothesis, as they consist of broadband neural activity (low-frequency LFP as well as high-frequency AMUA) including higher frequencies than the stimulus presentation rate, they peak at approx. 100–150 ms after an expected stimulus is omitted, and this latency is about 50 ms longer than the latency of the response to the presented stimulus.

### Omission responses along the auditory hierarchy

While omission responses have been reported at very early processing stages in the visual system [54], invasive and non-invasive studies suggest that auditory omissions are typically only found in the cortex [27, 28, 32, 33, 36, 37], except one study using intracellular recordings that found omission responses in the non-lemniscal thalamus [35]. Omission responses have not been found in the inferior colliculus [55] or in the brainstem [56]. 

Based on our recordings in the auditory cortex, we observed that omission response amplitude was correlated with evoked response amplitude across channels, suggesting that the same areas that encode sounds might also signal sound omissions. Indeed, we found that channels were mostly sensitive to either sound alone (25–39% of analysed channels) or to both presented and omitted sounds (33–55% of analysed channels), and that very few channels (4–10%) were found to be sensitive to omissions alone. This is in contrast to a recent study [26] that used relatively low-spatial resolution surface electrodes to measure broad local field potentials from the human superior temporal gyrus. The previous study reported neuronal populations that responded only to omitted sounds, but not to presented sounds [26]. This difference may be due to the surface electrode picking up more responses from higher auditory areas than in our study. While we did not collect post-mortem histology data to allow for precise subfield localization, the deliberate targeting of most of our penetrations to the auditory core by anatomical landmarks and the latencies of the noise responses of channels suggest the majority of our electrode shanks were in core regions of auditory cortex (see Methods). Taken together, these results are consistent with studies examining mismatch responses to deviant sounds, which found that mismatch-specific responses are more pronounced in hierarchically higher regions of the auditory pathway [13, 57, 58]. Future studies should test whether a similar gradient might be observed for omission responses.

### The implications of robust omission responses in field potentials rather than spikes

One point for consideration is that while we see omission responses in the LFP and the AMUA, we do not see them in the single-unit or multi-unit activity. Although our choice of stimulus (noise bursts, rather than e.g. pure tones) might have influenced the observed responses, a recent study in humans has found omission responses also to entirely unpredictable stimuli [24], consistent with the hypothesis that omitted noise bursts should also yield omission responses. Furthermore, given the relatively low number of penetrations and animals in the current study, as well as their anaesthetised state, we cannot exclude the possibility that future studies will indeed find evidence for omission responses in spiking activity [59]. Nevertheless, our study does show that omission-related signals are much more robust in the LFP and the AMUA. This may have implications for the representation of prediction and prediction error.

First, we must consider what the LFP and AMUA represent. LFP is considered to represent the dendritic activity of neurons, likely from summed inputs but perhaps also from dendritic processing [39, 40]. The AMUA, while typically taken to represent the summed output spiking activity, may perhaps have some contributions from other neural potentials such as incoming spikes and postsynaptic potentials. For example, in the auditory cortex, the correlation is only ~ 0.6 between turning curves from the spiking activity and the AMUA [60], and also, simulations suggest there is some power in massed excitatory postsynaptic potentials that is in the AMUA range, above 300 Hz [39]. Indeed, recent work suggests that high spike rates in the neocortex tend to correlate with field potential oscillations in the 50–180 Hz range, and less so with the lower range of LFPs or the higher range of the AMUA [61].

Our results suggest a number of non-exclusive possibilities. (1) Omission responses are present in the spike responses of neurons but only a small fraction of them. In our sample of 113 single- and multiunits, none showed a notable emission response, indicating that this fraction, if it exists, may be small. Furthermore, if this alone is the source of omission responses, it is hard to explain the size of the omission responses in the LFP and AMUA, being about half the size of the response to the presented stimuli. One possibility is that this fraction of neurons is clustered at a particular layer or region in the auditory cortex that we did not sample sufficiently. Our sample of single- and multi-units spans the depth of the cortex and we did not see units responding to omissions at any depth. It is also possible that the fraction could grow under non-anaesthetised conditions, which should be addressed in future studies. (2) Omission responses in spikes only occur for certain stimulus conditions. For example, omission responses in the original human EEG studies were described at faster presentation rates than the ones tested here [17, 62], which was interpreted as evidence for a particular window of integration of incoming auditory information. It is therefore possible that spiking omission responses may only be observed at faster presentation rates. (3) A third possibility is that the neurons whose spikes signal the omission response are from regions outside the auditory cortex, and they synapse on auditory cortical neurons and alter their membrane potentials but this does not in turn impact the auditory cortical neurons’ spiking activity. This implies that the relevant resulting potentials are sub-threshold and perhaps somewhat isolated from the soma in a distant region of the neuron’s dendrites. For example, cortical apical dendrites are somewhat electrotonically isolated from the site of spike generation at the axon hillock of the soma [63, 64]. (4) A fourth possibility is that the omission responses are calculated in the neuron by the summation of excitatory and inhibitory potentials, but that likewise, this does not in turn impact the neurons’ spiking activity, again because it is sub-threshold and also perhaps isolated in the apical dendrites or other dendrites.

This paucity of neural spiking omission responses, accompanied by a strong field potential omission response, has implications for models of the cortex. It calls into question models which require many neurons whose spike-output signals prediction or prediction error, although it could be that such models could operate for timescales or situations other than those that we examined. Our findings may be somewhat congruent with recent modelling work which proposed that prediction error or related signals are represented in the dendrites of pyramidal neurons; either in the apical dendrites [65–69], basal dendrites [70–72] or both [73–76]). These dendritic signals have been proposed to enable dendritic forms of hierarchical predictive coding [73] or backpropagation-like credit assignment [65, 74–76]. Given the complex hierarchical networks of the brain, how the brain assigns credit signals (such as prediction error) to the appropriate neurons and synapses to enable learning, without interfering with ongoing neural processing, is a key problem in neuroscience known as the credit assignment problem [74]. It has been argued that the electrotonic isolation of the apical dendrites allows for the segregation of their proposed credit assignment calculations from the ongoing sensory integration at the soma and oblique and basal dendrites [74]. Finally, the paucity of spiking omission responses may also explain why stimulus omissions are not typically mistaken with actual stimuli, as one of the main factors differentiating stimulus-driven from internally driven processes is the strength and precision of low-level cortical activity [77].

## Conclusions

The representation of prediction or prediction error is important for the learning or inference processes of various general hypotheses of brain function [2–6]. Using electrophysical recordings from the anaesthetised rat auditory cortex, we report that cortical field potentials show ‘omission responses’ that occur when sound bursts are randomly omitted from steady trains of sound bursts. If the rate of the sound bursts is changed, the omission responses remain locked to the expected time of the omitted burst. Omission responses have not previously been seen in the auditory cortex at such fast rates (2–4 Hz) in animal models. These results are consistent with some dependence of cortical local field potentials on prediction or prediction error.

## Methods

### Auditory paradigm

Auditory stimuli were delivered binaurally using custom-built in-ear headphones. The stimuli consisted of trains of broadband noise bursts presented at a fixed (isochronous) rate of 2, 3, or 4 Hz (Fig. 1B). Each noise burst was 25 ms long and 80 dB SPL. The noise bursts were embedded in a background of continuous white noise, at a burst-to-background ratio of 10 dB. Each stimulus train was 40 s long, with the first 36 s containing noise bursts and the last 4 s containing only background noise. In each train, a pseudo-random subset of 5% bursts was omitted. In all but one experiment, each train started with at least 12 bursts with no omissions, and subsequent omissions were separated by at least 5 bursts. In the remaining experiment, omissions were implemented pseudo-randomly throughout the stimulus sequence (separated by at least 3 bursts). Per experiment, 90 trains were presented, divided into 9 blocks of 10 trains each. The noise burst rate did not change during each block. There were 3 blocks with all 2 Hz trains, 3 blocks with all 3 Hz trains and 3 blocks with all 4 Hz trains. Between blocks, the noise burst rate changed pseudo-randomly (two consecutive blocks could not have the same burst rate). The placement of omissions in the sequence differed across stimulus trains and blocks. The burst rates were different across experiments.

### Subjects and surgical procedures

All experimental procedures obtained approval and licences from the UK Home Office and followed legal requirements (ASPA 1986). The subjects were three female adult Lister hooded rats weighing 225–363 g (mean = 286 g) at the time of the experiment. Rats were anaesthetised with a mixture of 0.05 ml domitor (1 mg/ml) and 0.1 ml ketamine (100 mg/ml), administered intraperitoneally. To maintain anaesthesia, rats were infused continuously with a saline solution containing 16 µg/kg/h domitor, 4 mg/kg/h ketamine and 0.5 mg/kg/h torbugesic, at a rate of 1 ml/h. Body temperature was maintained with a heating pad at 36° ± 1 °C. The depth of anaesthesia was controlled by regular testing of the absence of a toe pinch withdrawal reflex. The anaesthetised rats were placed in a stereotaxic frame with hollow ear bars set to fix the head for craniotomy. A craniotomy was performed with a centre at 4.7 mm caudal to bregma and 3.5 mm lateral to the midline. In two rats (rats 1 and 3), the craniotomy was performed over the right hemisphere; in one rat (rat 2), the craniotomy was performed over the left hemisphere.

### Electrophysiological recordings and pre-processing

Electrophysiological data were recorded using a 64-channel silicon probe (Neuronexus Technologies, Ann Arbor, MI, USA) with 8 shanks each with 8 equally spaced electrodes along its length, forming a square grid pattern of 8 × 8 recording sites (electrode diameter: 175 µm^2^; distance between electrodes: 0.2 mm). Anatomical coordinates were used to position the probe over the auditory cortex. The probe was then inserted into the brain at a medio-lateral orientation until all recording sites were inside the brain. First, to verify that the recording sites were driven by sound stimulation, a search stimulus consisting of broadband noise bursts was played. Next, to check that channels contained signals from neuronal populations sensitive to acoustic frequency, frequency response areas (FRAs) were measured. Following these checks, experimental stimuli were presented binaurally via headphones at approximately 80 dB SPL. The stimulus sampling rate was set to 48,828.125 Hz. Electrophysiological data were acquired at a sampling rate of 24,414.0625 Hz using a TDT system 3 recording set-up (Tucker Davis Technologies). Across rats, data were recorded from 6 penetrations (rat 1: 1 penetration; rat 2: 3 penetrations; rat 3: 2 penetrations). The first penetration typically targeted the coordinates of the primary auditory cortex. In rats with multiple penetrations, the consecutive experiments were performed after moving the probe towards more rostral (rat 2) or dorsal (rat 3) sites by approx. 500 μm and repeating the search stimulus and FRA recordings. The response latency of analogue multi-unit activity (see AMUA analysis in the ‘Methods’ section) in response to noise bursts was assessed for each penetration. These were 13 ms for 3/6 penetrations; 20 ms for 2/6 penetrations, and 27 ms for 1 penetration. We also repeated this analysis per shank (rather than per penetration) and found the median response latency across all 48 shanks to be 17 ms (first quartile: 13 ms; third quartile: 27 ms). Compared with reported spike latencies in the rat auditory cortex [78], this suggests that the majority of our penetrations were in the core regions of the auditory cortex, including the primary auditory cortex, with one penetration potentially in a belt region.

Data traces (acquired in long segments of 40 s, corresponding to stimulus trains) were filtered off-line using 7th-order two-pass Butterworth filters: a notch filter (49–51 Hz) to remove line noise, and a high-pass filter (cut-off frequency: 0.1 Hz) to remove low-frequency drifts. Data were then epoched into shorter segments, corresponding to stimulus omissions (from − 100 ms to 250 ms relative to the onset of expected but omitted stimuli) and the immediately preceding noise bursts (from − 100 ms to 250 ms relative to burst onset). Segments were selected to end at 250 ms relative to the presented or expected burst onset, since at the fastest presentation rate (4 Hz) this is when the next burst is presented. The resulting number of omission and preceding burst epochs per penetration (mean ± SD) was 89 ± 4.89 for the 2 Hz burst rate; 165.66 ± 18.78 for the 3 Hz burst rate; and 232.16 ± 10.41 for the 4 Hz burst rate.

### Data analysis

#### Amplitudes and latencies of omission-evoked responses

Short segments were analysed in three ways, to obtain measures of local field potentials (LFP), analogue multiunit activity (AMUA), and single- and multiunit spiking activity. In the LFP analysis, low-frequency signals were derived from original data by low-pass filtering each short segment using a 3rd order two-pass Butterworth filter (cut-off frequency: 75 Hz) and downsampling to 150 Hz. To avoid signals from channels with different polarity cancelling each other out, LFP traces from channels showing a negative burst-evoked peak amplitude in the period 0–250 ms after noise-burst onset (averaged across presentation rates) were sign-flipped. In the AMUA analysis, data were band-pass filtered using a 3rd order two-pass Butterworth filter between 300 and 6000 Hz [79]. Then the analytic envelope (calculated using a 2-tap FIR filter) of the band-pass data was downsampled to 150 Hz. In both analyses, the epoched traces were normalised by *z*-scoring each trace relative to the pre-stimulus baseline (from -100 ms to 0 ms relative to burst onset, during the 70 dB background noise). To remove outliers, we calculated a standard deviation of the voltage fluctuation in each trial (SDi) and rejected trials with SDi beyond the median ± 3 SD of all SDi values. To quantify response latency, per penetration, we averaged AMUA responses across trials and burst presentation rates, and calculated the first latency relative to burst onset for which AMUA amplitude exceeded its half-maximum, relative to the pre-stimulus baseline.

To test whether single channels show noise-burst-evoked activity, single-trial amplitudes were averaged over time (from 0 to 250 ms relative to burst onset), pooled over burst rates, and (since the data were already normalised to the pre-stimulus baseline) entered into a one-sample t-test for each channel. Similarly, to test whether single channels show omission-evoked activity, amplitudes were averaged over time (from 0 to 250 ms relative to expected but omitted burst onset), pooled over burst rates, and subjected to one-sample *t*-tests. The resulting p-values were corrected for multiple comparisons using a false-discovery rate p_FDR_ < 0.05 [42]. Only those channels showing both significant burst- and omission-evoked responses were entered into subsequent analyses. Since pooling signals over time can result in peaks and troughs cancelling out (specifically in the case of LFP data), we quantified the peak-to-trough asymmetry for each channel by calculating the area under the curve (AUC) values separately for the positive (AUC +) and negative (AUC −) polarity, and dividing their absolute difference by their sum. The resulting asymmetry index approaches 0 for perfect AUC + /AUC − symmetry and 1 for purely positive/negative polarity of the signal. Among the selected channels, the median asymmetry indices of omission LFP responses were 0.98 and 0.97 for selected and non-selected channels respectively, suggesting that our selection criterion does not bias the response of omission responses. However, in a control analysis, we also analysed data from all channels, without any selection criteria (Fig. 3A). Single-trial data from the selected channels were averaged per penetration, channel, burst rate (2, 3, and 4 Hz), and stimulus type (burst vs. omission). Average traces were used to extract peak amplitudes and latencies.

Omission-evoked responses were compared with burst-evoked responses using mixed-effects modelling. In separate analyses, we compared peak amplitudes and peak latencies. In both cases, single-channel data were entered into a mixed-effects model with two fixed-effects factors (stimulus type: burst vs. omission; burst rate: 2, 3, and 4 Hz) and one random-effects factor (penetration). In a control analysis, to test for the possibility that omission-related activity is due to random noise fluctuations in the post-omission time window rather than to true omission responses, we repeated the analysis described above but after shuffling single-trial data over time points (over -100 to 250 ms; Fig. 3B).

To test whether omission responses build up over time (as a function of the number of preceding noise bursts), for each trial and analysed channel we extracted the peak amplitude of the omission response, averaged these peak amplitudes across channels, and correlated them with the log number of preceding noise bursts to model exponential decay [43]. The Pearson correlation analysis was conducted separately for each signal type (LFP, AMUA) and burst rate (2, 3, and 4 Hz). Significance of correlation coefficients was Bonferroni-corrected for multiple comparisons.

Finally, in the analysis of spiking activity, we performed offline spike sorting using the expectation–maximization algorithm Klustakwik [80] followed by manual post-processing using the Klustaviewa toolbox (Cortical Processing Lab, University College London). The algorithm returns two types of clusters of spikes—one putatively originating from a single neuron (termed a single unit, *n* = 79 across 6 penetrations) and one putatively originating from a small population of neurons (termed a multiunit, *n* = 280) near a recording site. Firing rate time series were calculated by binning spike times into 10 ms bins, resulting in peri-stimulus time histograms (PSTHs) sampled at 100 Hz. Only those single units and multiunits that were reliably driven by stimuli (noise bursts) were included in further analysis. In order to quantify firing reliability, we used a noise power to signal power metric [81], which characterises the repeatability of neural response patterns for multiple presentations of the same stimulus. Neural responses to the first 250 ms of all 3 burst rates (2, 3, and 4 Hz) were pooled for this analysis. Following previous studies [82, 83], only those units showing a noise power ratio higher than 40 were included in the analysis, amounting to 43 single units (54.43%) and 70 multiunits (25%).

PSTHs were analysed to test for significant omission-evoked responses. Baseline spontaneous firing rate (SFR) was quantified as the average firing rate during the 50 ms preceding noise burst onset. To generate a null distribution, 1000 simulated PSTHs were calculated using a Poisson model assuming a constant firing rate equal to the SFR [13]. For both actual and simulated PSTHs, response amplitudes were baseline-corrected by subtracting the SFR. Post-omission baseline-corrected PSTHs were tested for statistical significance by calculating the p-value of the actual PSTH as *p* = (*k* + 1)/(*N* + 1), where *k* is the count of simulated PSTHs for which the root mean square (RMS) over the post-stimulus period (0–250 ms, averaged across burst rates) was greater than or equal to the RMS of the actual PSTH, and *N* = 1000 simulations. This procedure could yield a minimum *p* ≈ 0.001. The resulting p-values were corrected for multiple comparisons using a false-discovery rate p_FDR_ < 0.05 [42].

#### Cortical depth of omission-evoked responses

To test whether omission responses preferentially engage cortical activity in relatively superficial or deep channels and whether the cortical depth profile depends on the type of responses (higher-frequency/AMUA vs. lower-frequency/LFP), we analysed peak amplitudes at electrodes which yielded significant omission responses of both types (AMUA and LFP). In order to increase the depth resolution of the LFP signals, they were converted to current source density (CSD) estimates by calculating the second spatial derivative over channels. Given that each shank contained 8 electrodes, this procedure resulted in 6 CSD estimates as a function of channel depth. To make AMUA and CSD data more comparable, we therefore removed the edge channels from AMUA analysis. Data were pooled across penetrations, and electrode shanks into three groups of channels: superficial (channels 2–3 of each shank), intermediate (channels 4–5), and deep (channels 6–7). The same criteria were applied to assigning single units and multiunits (SUA, MUA) to different cortical depths, except for edge channels being included (superficial: channels 1–3; intermediate: channels 4–5; deep: channels 6–8). To summarise the relative strength of omission responses, we calculated the omission response index of each channel (i.e. the peak amplitude of the omission response divided by the average peak amplitude of the omission and burst-evoked response). The resulting omission response index was always positive, lower than 1 if the omission response was weaker than the burst-evoked response, and higher than 1 if the omission response was stronger than the burst-evoked response. Omission response indices were compared between response types (AMUA vs. CSD) and channel groups (superficial, intermediate, deep) in a 2 × 3 ANOVA across channels. Post hoc two-sample *t*-tests (separate for each channel group) were corrected for multiple comparisons using Bonferroni correction.

## Data Availability

All data generated or analysed during this study are included in this published article and supplementary information files. The datasets supporting the conclusions of this article are available in the https://gin.g-node.org repository, doi:10.12751/g-node.fedc41.

## Abbreviations

AMUA: Analogue multiunit activity
ANOVA: Analysis of variance
AUC: Area under the curve
CSD: Current source density
EEG: Electroencephalography
FRA: Frequency response area
ISI: Interstimulus interval
LFP: Local field potential
MEG: Magnetoencephalography
MUA: Multiunit activity
PSTH: Peristimulus time histogram
RMS: Root mean square
SD: Standard deviation
SEM: Standard error of the mean
SFR: Spontaneous firing rate
SPL: Sound pressure level
SUA: Single unit activity
